# Medical Students’ Attitudes and Experiences Regarding Persuasion of Patients by Physicians: Clarifying the Ethics of Shared Decision Making

**DOI:** 10.1007/s11606-025-09807-w

**Published:** 2025-09-04

**Authors:** John Muckler, James C. Thomas, Laura Shinkunas, Lauris C. Kaldjian

**Affiliations:** 1 Carver College of Medicine, University of Iowa, Iowa City, IA USA; 2 College of Public Health, University of Iowa, Iowa City, IA USA; 3 Program in Bioethics and Humanities, Carver College of Medicine, University of Iowa, Iowa City, IA USA; 4 Department of Internal Medicine, Carver College of Medicine, University of Iowa, 500 Newton Road, 1-106 MEB, Iowa City, IA 52242 USA

**Keywords:** persuasion, shared decision making, ethics, medical students, physicians

## Abstract

**Background:**

Ethical principles of autonomy and beneficence guide clinical decision making. Little is known about how clinicians prioritize these principles and integrate them with virtue ethics when assessing the ethics of persuasion.

**Objective:**

Survey medical students about their attitudes and experiences regarding the use of persuasion by physicians in shared decision making.

**Design:**

Cross-sectional, on-line survey.

**Participants:**

Pre-clinical and clinical medical students at one US medical school.

**Main Measures:**

Survey instrument contained 31 items including a three-part clinical vignette, attitudes toward persuasion and ethical principles, experiences observing or participating in persuasion, and demographic information. Bivariate and multivariable analyses were performed, including LASSO regression using a 30-point persuasion score derived from six items.

**Key Results:**

237 students completed the survey (45% response rate). In general, 55.7% supported persuasion by physicians for the good of the patient’s health. Nearly half supported persuasion for an at-risk cardiovascular patient who declines recommendations for walking or statin treatment; 64.1% supported persuasion for a patient with myocardial infarction who wants to leave the hospital against medical advice. While 70.0% believed persuasion is appropriate because it promotes beneficence and nonmaleficence, 16.9% believed persuasion is inappropriate because it disrespects patient autonomy. Most students (81.0%) had seen a good physician role model for persuasion, and 38.0% had willingly participated in persuasion. LASSO regression identified four items contributing positively to the persuasion score (belief that persuasion promotes beneficence/nonmaleficence, observation of a good role model, experience participating in persuasion, male gender) and two negatively (belief that persuasion disrespects patient autonomy, observation of inappropriate use of persuasion).

**Conclusions:**

Medical students vary in attitudes toward persuasion of patients by physicians, and variations are associated with differences in ethical beliefs, clinical experiences, and gender. Education regarding the use of persuasion should address ethical justification, experience, and role-modeling—which can be encompassed by virtue ethics.

**Supplementary Information:**

The online version contains supplementary material available at 10.1007/s11606-025-09807-w.

## INTRODUCTION

Shared decision making is the preferred approach to decision-oriented communication in clinical practice, consistent with informed consent and respect for patient autonomy.^1–3^ It represents a partnership between patients and clinicians when deliberating about treatments and tests. For some, shared decision making applies when more than one reasonable option is available, in contrast to motivational interviewing, which applies when a patient is ambivalent about a medically indicated behavior change that is desired by the patient.^4,5^ Such contrasts illustrate the challenge of defining shared decision making^6^ in ways which include clinician guidance, patient values and preferences, and “an interpersonal, interdependent process in which the health care provider and the patient relate to and influence each other as they collaborate in making decisions about the patient’s health care.”^7^ The extent of the physician’s influence, and whether it should sometimes encompass persuasion,^8^ is the topic of this study.

Persuasion in healthcare can be understood as involving the intentional effort to change a patient’s beliefs, position, or course of action based on the strength of reasons offered by a clinician.^9,10^ While *informing* patients and giving *recommendations* are commonly accepted in shared decision making and consistent with ethical and legal expectations,^11–13^ *persuading* patients is less often discussed, even though persuasion commonly occurs in situations of disagreement, such as in the care of patients who act against medical advice^14^ or request treatments perceived as futile or potentially inappropriate.^15^ Concerns related to the ethics of persuasion may also be found in discussions about the ethics of nudging—i.e., communications that influence patients’ choices in predictable directions^16^ through non-rational processes.^17^ Nudging contrasts with reasoning-based persuasion by exploiting patients’ cognitive biases instead of appealing to their rational capacity for goal-directed deliberation.^18^

At first glance, persuasion may seem to contradict patient autonomy in shared decision making. However, we agree with those who think that its use can be defended by principles of beneficence and nonmaleficence,^19^ if persuasion is seen as promoting the patient’s good by encouraging options that are more beneficial and less harmful. Persuasion can be seen as standing in the middle of a spectrum that moves from appropriate degrees of influence (informing and recommending) on one side, to inappropriate degrees of influence (manipulating or coercing) on the other.^20^ Moreover, patient autonomy itself can be reconciled with persuasion if autonomy is understood not merely as choice,^21^ but as a capacity (“relational autonomy”^22,23^) promoted by supportive relationships. And while we agree that attempts at persuasion are sometimes ethically justifiable by principles in theory, in practice we acknowledge that knowing when and how to attempt persuasion in shared decision making is a serious professional responsibility which requires practical wisdom and other virtues.^24–26^ This “knowing when and how” draws attention to the relevance of virtue ethics for healthcare and the need to integrate principles, virtues, and consequences within a coherent bioethical framework.^27^

Persuasion in shared decision making has received conceptual analysis and some empirical studies of patients and physicians,^28–30^ but there appears to be no published research of medical students’ attitudes toward the use of persuasion by physicians. To address this gap, we performed a cross-sectional survey of pre-clinical and clinical medical students about their attitudes toward shared decision making and the use of persuasion by physicians. We sought to determine whether students’ views are associated with differences in (1) level of exposure to clinical practice, (2) observation of or participation in persuasion, (3) ethical principles, and (4) demographic variables.

## METHODS

### Participants and Setting

We conducted a cross-sectional, anonymous, online survey at the University of Iowa Carver College of Medicine (CCOM), an allopathic medical school which admits approximately 152 students each year. The 4-year curriculum comprises preclinical (1.5 years) and clinical (2.5 years) phases, such that by May/June of each year, M1 students have completed 1 year of preclinical education, M2 students have completed 1.5 years of preclinical education and approximately 0.5 years of clinical education, and M3 students have completed approximately 1.5 years of preclinical education and 1.5 years of clinical education. Surveys were distributed to 525 medical students in the first (M1), second (M2), and third (M3) years of medical school, as well as MD-PhD (Medical Scientist Training Program (MSTP)) students at equivalent points in their education.

In the pre-clinical portion of the curriculum, the CCOM’s “Biomedical Ethics Thread” highlights three theoretical approaches (principles, virtues, consequences) and includes 11 recorded lectures, each of which is followed by an in-person, case-based, large group discussion. One of these lecture/discussion sessions in the M1 year (first semester) is “Informed Consent and Shared Decision Making.” This introduces students to the ethical rationale for informing, recommending, and sometimes persuading; the tension between autonomy and beneficence inherent in persuasion; the concept of relational autonomy; and the ethical management of patient-clinician disagreements (including “against medical advice” hospital discharges) with the potential need for respectful attempts at persuasion.

### Survey Instrument Development

The survey instrument (Appendix) contained 31 questions, including a clinical vignette with three separate scenarios. Definitions of key terms were provided: “inform” (to impart information or knowledge); “recommend” (to suggest an act or course of action as advisable); “persuade” (to move by argument to a belief, position, or course of action); “persuasion in healthcare” (persuasion is attempted when, after having communicated information and a recommendation, a healthcare professional uses reasoning to try to change a patient’s thinking toward a decision that is more consistent with the healthcare professional’s understanding of what is good for the patient’s health).

The three scenarios of the clinical vignette posed the question of persuasion in response to a patient who declines a recommendation for (1) **walking**, to decrease the risk of heart disease and early death; (2) **taking a statin medication** because of a very high cardiovascular risk score, to decrease the risk of having a potentially deadly heart attack; and (3) **staying in the hospital** for treatment of a myocardial infarction, to decrease the risk of a serious medical complication, including the possibility of sudden cardiac death. These three scenarios, involving potentially negative outcomes entailing increasing risks of harm, were designed to (1) demonstrate that the physician was trying to act in the patient’s best interests; (2) indicate that the physician was trying to persuade the patient (i.e., doing more than just recommending a course of action); and (3) pose the question of whether or not persuasion was ethically appropriate under the circumstances. Response options used a 5-point Likert scale (strongly agree, agree, not sure, disagree, and strongly disagree).

The survey instrument also asked students about their general attitudes toward aspects of shared decision making (informing, recommending, and persuading), ethical principles, and concepts of health, using the same 5-point Likert scale response options. Students were also asked about their experiences observing and participating in attempts at persuasion, with response options of yes, no, and unsure. Lastly, students were asked to provide demographic information (year in medical school; anticipated medical specialty; age, gender; race/ethnicity; state residency status).

The survey instrument was pilot tested by three students (M1, M2, M3) and refined to enhance clarity.

### Survey Administration

From May 14 to June 19, 2024, we emailed the survey to 525 medical students at the end of the M1, M2, or M3 years of medical school. An initial invitation was followed by four reminder emails to non-responders. The survey used Qualtrics (Provo, UT) and took 10–15 min to complete. Participants were required to answer all questions to receive a $10 gift card after completion. The study and its informed consent process were approved by the University of Iowa’s institutional review board (IRB ID # 202404397).

### Statistical Analysis

Survey responses were downloaded from Qualtrics to Microsoft Excel to tabulate frequencies and imported into R (R Core Team, 2023) for bivariate analysis and regression modeling. There were no missing data except for responses to two demographic questions which gave the option of “prefer not to say”: gender (7 missing) and race/ethnicity (12 missing). Likert scale responses were dichotomized as agree/strongly agree vs neutral/disagree/strongly disagree (combining neutral and negative responses placed primary analytic focus on positive responses). To simplify reporting in the Results, ***agree*** signifies the combination of “agree” and “strongly agree” responses. For inter-year comparisons of students’ education levels, we combined groups as follows: M1 combines “pre-clinical: summer between M1/M2 years” and “MSTP-preclinical”; M2 combines “clinical: summer between M2/M3 years” and “MSTP-fewer than 6 months of clinical rotations”; and M3 combines “clinical: summer between M3/M4 years” and “MSTP-more than 6 months of clinical rotations.” Regarding students’ anticipated specialty choices, we compared primary care vs non-primary care specialties^31^ and surgical vs non-surgical specialties.^32^ Bivariate associations used the Pearson’s Chi-squared test, or Fisher’s exact test when sample size dictated. Associations were considered significant at an alpha level of 0.05.

To analyze multivariable relationships, six of the 5-point Likert scale questions were aggregated into a measure named “persuasion score”. Thus, the persuasion score had a minimum possible value of 5 and a maximum possible value of 30. The six questions aggregated to create the persuasion score were: (1) “In general, a physician should attempt to persuade a patient…”; (2) clinical vignette regarding persuasion about walking; (3) clinical vignette regarding persuasion about a statin medication; (4) clinical vignette regarding persuasion about staying in the hospital; (5) “… as a medical student I am willing to participate in attempts to persuade…”; and (6) “When I am a physician, I expect to feel a responsibility to attempt to persuade.…” The persuasion score served as the dependent variable representing students’ overall attitude toward persuasion. This score was regressed on the rest of the survey questions using the LASSO regression^33^ implemented in the glmnet R package.^34^ LASSO regression automatically performs variable selection, which is desirable in the presence of many potential independent variables. The result is a parsimonious and easily interpreted model with all variables unimportant to the relationship removed. Variables that remain in the model after the LASSO regression are considered to have an important impact on persuasion score.

## RESULTS

### Response Rate and Demographic Characteristics

The survey was completed by 237 medical students (response rate: 45.1%). As shown in Table1, participants were distributed evenly across class years; had a mean age of 25.3 years; were more commonly female, Caucasian/White, and in-state residents; and less commonly interested in primary care (vs non-primary care) or surgical (vs non-surgical) specialties. Regarding their concepts of health, 113 (47.7%) of students agreed that health is an objective biological condition, and 153 (64.6%) agreed that health is a subjective perception of well-being.

**Table 1.** Demographic Characteristics of 237 Participants

Education level, *n* (%)	
M1: first-year students	86 (36.3)
M2: second-year students	73 (30.8)
M3: third-year students	78 (32.0)
Age, years	
Mean	25.3
Median	25
Range	21–42
Gender, *n* (%)	
Female	134 (55.6)
Male	96 (39.8)
Non-binary/non-conforming	3 (1.2)
Transgender	1 (0.4)
“Prefer not to say”	7 (2.9)
Race/ethnicity, *n* (%)	
Caucasian/White	172 (62.8)
Asian	45 (16.4)
Hispanic/Latino	19 (6.9)
South Asian	11 (4.0)
Black/African-American	6 (2.2)
Middle Eastern/North African	4 (1.5)
American Indian/Alaskan Native	3 (1.1)
Native Hawaiian/Pacific Islander	1 (0.4)
Other	1 (0.4)
“Prefer not to say”	12 (4.4)
Residency status,* n* (%)	
In-state (Iowa)	162 (68.4)
Out-of-state	73 (30.8)
Other	2 (0.8)
Anticipated specialty choice, *n* (%)	
Primary care specialties	79 (33.3)
Surgical specialties	75 (31.6)

### Attitudes and Experiences

As shown in Table 2, in general, almost all students agreed that a physician should inform patients and make recommendations, and 55.7% supported the use of persuasion. In response to the clinical vignette scenarios, nearly half of the students agreed with the use of persuasion for walking (48.9%) or for a statin medication (47.7%), and 64.1% agreed with persuasion to stay in the hospital for treatment of a myocardial infarction. Only 16.9% of students believed persuasion is inappropriate (because it disrespects patient autonomy), whereas 70.0% believed it is appropriate (because it promotes beneficence and nonmaleficence); 48.1% were willing to participate as students in attempts to persuade patients, and 70.5% expected to feel a responsibility to attempt to persuade patients after becoming a physician.

**Table 2:** Students’ Attitudes and Experiences

Attitude	Agree *n* (%)
In general, a physician should …	
** … inform** a patient about treatment options ** … make recommendations** to a patient about treatment options ** … attempt to persuade** a patient when a patient does not accept a recommendation the physician believes is good for the patient’s health	231 (97.5) 227 (95.8) 132 (55.7)
Clinical vignette scenarios: It is **ethically appropriate for the physician to attempt to persuade** the patient to change his decision about …	
** … walking** to decrease risk of heart disease and early death ** … taking a statin medication** because of a very high cardiovascular risk score ** … staying in the hospital** for treatment of a myocardial infarction	116 (48.9) 113 (47.7) 152 (64.1)
Persuasion by healthcare professionals is …	
…ethically ***inappropriate*** because it **disrespects patient autonomy** …ethically ***appropriate*** because it **promotes beneficence and nonmaleficence**	40 (16.9) 166 (70.0)
Within the scope of my responsibility **as a medical student**, I am willing to participate in attempts to **persuade** patients who decline recommendations made by the healthcare team	114 (48.1)
** When I am a physician**, I expect to feel a responsibility to attempt to **persuade** patients to change their minds if they decline a treatment which I think would be good for their health	167 (70.5)
Experience: Observation	Yes *n* (%)
I have observed (at least once) a physician …	
… **attempt to use persuasion *appropriately*** with a patient or a patient’s decision maker … **attempt to use persuasion *inappropriately*** with a patient or a patient’s decision maker … who, in my opinion, **should have attempted to use persuasion** (but did not) when a patient refused a recommended treatment	208 (87.8) 73 (30.8) 82 (34.6)
I have observed at least one physician …	
… who was **a good role model** for how to use **persuasion** with a patient in a way that is respectful and clear about the intention to influence the patient’s decision … **try to influence** a patient’s treatment decision **without making it clear** to the patient that they were trying to influence the patient’s decision	192 (81.0) 106 (44.7)
Experience: Participation	Yes *n* (%)
Within the scope of my responsibility as a medical student …	
… I have **willingly participated** (at least once) in an ***appropriate*** attempt to **persuade** a patient who was refusing a treatment the healthcare team was recommending … I have **felt compelled** to participate (at least once) in an ***inappropriate*** attempt to **persuade** a patient who was refusing a treatment the healthcare team was recommending	90 (38.0) 14 (5.9)

Table 2 also shows that 87.8% of students had seen persuasion used appropriately; 81.0% had seen a good physician role model for persuasion; and 34.6% had observed a situation in which a physician should have used persuasion but did not. By contrast, 30.8% had seen persuasion used inappropriately, and 44.7% had seen a physician try to influence a patient’s decision without being transparent. Regarding participation, 38.0% of students indicated they had willingly participated in an appropriate attempt to persuade a patient, and 5.9% reported they had felt compelled to participate in an inappropriate attempt to persuade a patient.

### Associations Between Attitudes and Beliefs About Ethical Principles and Concepts of Health

**Figure 1:**
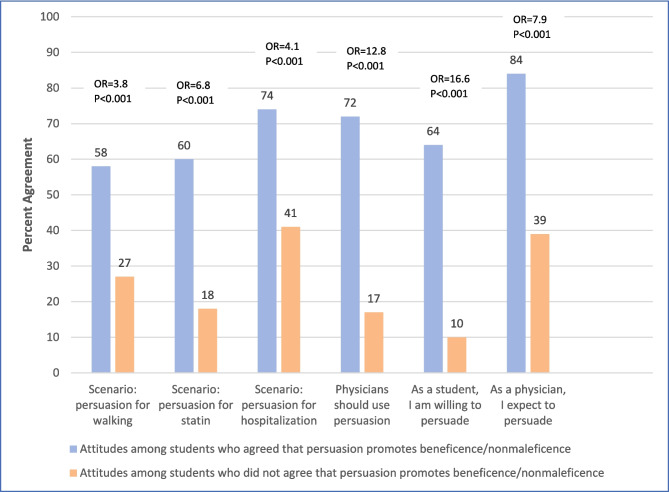
Attitudes toward using persuasion based on beliefs about beneficence/nonmaleficence.

Bivariate analyses in Fig. 1 show that students who agreed that persuasion is appropriate because it promotes beneficence and nonmaleficence were more likely to support the use of persuasion in the walking scenario, statin scenario, and hospitalization scenario, and more likely to believe that physicians should be willing to use persuasion in general. These students were also more likely to indicate a willingness to participate in persuasion as medical students and to expect to feel a responsibility to use persuasion as physicians in the future.

**Figure 2:**
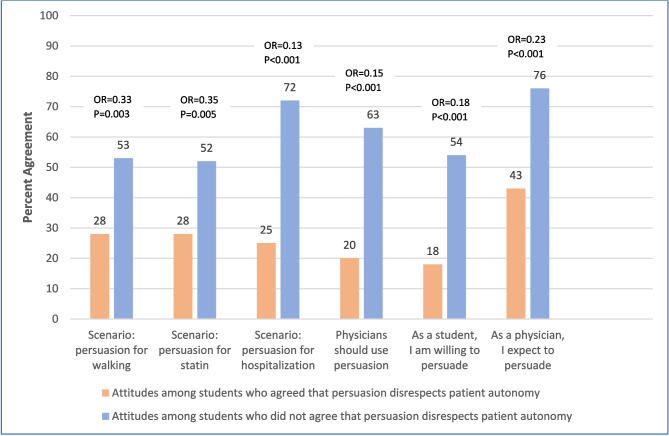
Attitudes toward using persuasion based on beliefs about patient autonomy.

By contrast, bivariate analyses in Fig. 2 show that students who agreed that persuasion is inappropriate because it disrespects patient autonomy were less likely to support the use of persuasion in the walking scenario, statin scenario, and hospitalization scenario, and less likely to believe that physicians should be willing to use persuasion in general. These students were also less likely to indicate a willingness to participate in persuasion as medical students or to expect to feel a responsibility to use persuasion as physicians in the future.

Bivariate analyses also showed that students who agreed that health depends primarily on an individual’s objective biological condition were more likely to believe physicians should be willing to use persuasion in general (64% vs 48%, OR = 1.87, *P* = 0.018). By contrast, students who agreed that health depends primarily on an individual’s subjective perception of well-being were less likely to believe physicians should be willing to use persuasion in general (50% vs 65%, OR = 0.53, *P* = 0.025).

### Associations Between Attitudes and the Experience of Observing a Good Physician Role Model

**Figure 3:**
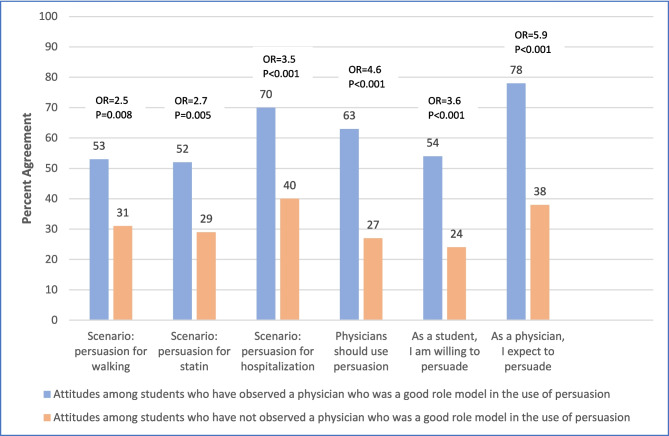
Attitudes toward using persuasion based on the experience of having observed a physician who was a good role model in the use of persuasion.

As shown in Fig. 3, bivariate analyses demonstrated that students who had observed a physician who was a good role model in the use of persuasion were more likely to support the use of persuasion in the walking scenario, statin scenario, and hospitalization scenario, and more likely to believe that physicians should be willing to use persuasion in general. These students were also more likely to indicate a willingness to participate in persuasion as medical students and to expect to feel a responsibility to use persuasion as physicians in the future.

### Associations Between Attitudes and Gender

Female students were less likely than male students to support the use of persuasion in the walking scenario (38% vs 65%, OR = 0.32, *P* < 0.001), statin scenario (38% vs 62%, OR = 0.37, *P* < 0.001), and hospitalization scenario (58% vs 74%, OR = 0.50, *P* = 0.017), and less likely to believe that physicians should be willing to use persuasion in general (51% vs 65%, OR = 0.55, *P* = 0.030). They were also less likely to indicate a willingness to participate in persuasion as medical students (43% vs 58%, OR = 0.55, *P* = 0.029).

### Regression Modeling Using a Persuasion Score

LASSO regression modeling results are shown in Table 3. In the model, 20 survey items (excluding the six questions used to create the persuasion score) were considered as potential independent variables: four items were found to contribute positively to the persuasion score, and two negatively. The persuasion score had a minimum possible value of 5 and a maximum possible value of 30. The item “persuasion … promotes beneficence and nonmaleficence” had the highest coefficient estimate (4.08), indicating that the response to this question had the most impact on the persuasion score among the Likert-style items (a student who agrees with this statement about persuasion is expected to have a persuasion score 4.08 points higher than someone who disagrees with it). The item “persuasion … disrespects patient autonomy” had a large but negative coefficient estimate (− 2.81), indicating that a student who agrees with this statement is expected to have a persuasion score 2.81 points lower than someone who disagrees with it. Students’ experiences of observing good role models of persuasion and participating willingly in persuasion had comparatively smaller estimated positive effects on persuasion score (0.68 and 0.42, respectively), while observing inappropriate attempts of persuasion had a small estimated negative effect of − 0.51. For the gender variable, we designated females as the reference group and found that males are expected to have a persuasion score 1.03 points higher than females.

**Table 3:** LASSO Regression Modeling Using a Persuasion Score

Variable	Reference Group	Estimate
(Intercept)	–-	16.85
Persuasion by healthcare professionals is ethically ***appropriate*** because it **promotes beneficence and nonmaleficence**	Disagree	4.08
Gender, Male	Female	1.03
I have observed at least one physician who was **a good role model** for how to use **persuasion** with a patient in a way that is respectful and clear about the intention to influence the patient’s decision	No	0.68
Within the scope of my responsibility as a medical student I have **willingly participated** (at least once) in an ***appropriate*** attempt to **persuade** a patient who was refusing a treatment the healthcare team was recommending	No	0.42
Persuasion by healthcare professionals is ethically ***inappropriate*** because it **disrespects patient autonomy**	Disagree	− 2.81
I have observed (at least once) a physician **attempt to use persuasion *inappropriately*** with a patient or a patient’s decision maker	No	− 0.51

## DISCUSSION

The results of this survey suggest that medical students vary in their attitudes toward the use of persuasion by physicians, and that their attitudes are associated with differences in ethical beliefs, clinical experiences, and gender. To our surprise, variations in their attitudes were not statistically associated with differences in class year, suggesting that the simple duration of clinical training is not as consequential as clinical experiences, ethical beliefs, and personal characteristics.

The ethics of persuasion involves the challenge of reconciling the imperatives of autonomy, on the one hand, with beneficence and nonmaleficence, on the other. These are three of the four principles of biomedical ethics,^9^ and their tensions demonstrate how principles are easier to discuss as abstractions than to apply in the concrete circumstances of clinical experience. The ethical challenge of persuasion arises when clinicians want to respect a patient’s self-determination while promoting the patient’s good, or best interests. Clinicians may conclude that the only way to resolve this tension is to prioritize one principle over another, whether that be beneficence^23,35^ or autonomy.^36^ As shown in our regression modeling, students’ ethical attitudes toward persuasion were notable contributors to their persuasion score.

Regarding clinical experiences, students who observed a positive role model of persuasion had a higher persuasion score, and students who observed a physician attempt persuasion in an inappropriate way were more likely to have a lower persuasion score. These findings suggest that in watching physician preceptors, students not only gain medical knowledge and skills but also learn what it means to care for patients ethically. These results confirm the significance of role-modeling in the formation of students’ ethical attitudes toward challenging aspects of clinical care.^37,38^

The moral impact of clinical encounters during training draws attention to the way students’ experiences contribute to the cultivation of their ethical understanding, moral agency, and professional identity, as understood within a framework of virtue ethics.^26,39^ When students reflect on their ethically significant clinical experiences, they participate actively in strengthening their virtues over time.^24,25,40^ Virtues that are important in healthcare generally are also relevant to the ethics of persuasion—virtues such as practical wisdom, patience, respectfulness, courage, compassion, diligence, humility, self-control, and integrity. These can be encouraged or discouraged by positive or negative clinical experiences involving persuasion.

Our study had limitations. The generalizability of our results is limited by the collection of data from only one medical school. We did not gather information about survey non-responders. Even though the survey was anonymous, social desirability bias may have led some students to give answers perceived as more professionally acceptable, and unmeasured differences among the class cohorts may have affected inter-class comparisons of attitudes. Lastly, we did not compare students’ attitudes with those of resident or attending physicians.

This study highlights the need for medical education about shared decision making, which includes the ethics of persuasion, especially during clinical rotations when students are likely to be exposed to both appropriate and inappropriate examples of persuasion. Discussing the ethics of persuasion is broadly relevant to the ethics of the patient-clinician relationship by encouraging candor about how beliefs and values influence clinical recommendations,^41,42^ by emphasizing the need for reason-based and respectful approaches to communication,^8^ and by illustrating the wisdom of relational autonomy.^22,23^ When such teaching is accompanied by role-modeling and experience-based reflection, students are well-positioned to cultivate the virtues needed to navigate tensions that arise in patient care when principles of autonomy, beneficence, and nonmaleficence require prioritization and integration. Such virtues are the moral character strengths that allow us to recognize why, when, and how to use persuasion, and to do so in ways that respect patients as persons and promote their good.

## Supplementary Information

Below is the link to the electronic supplementary material.

Supplementary file1 (PDF 225 KB)
