# Supplementary material for: Medical Students’ Attitudes and Experiences Regarding Persuasion of Patients by Physicians: Clarifying the Ethics of Shared Decision Making
Source: J Gen Intern Med. 2025 Sep 4;40(16):3870–8. doi: 10.1007/s11606-025-09807-w (PMC12686280; doi:10.1007/s11606-025-09807-w)
Supplement: Supplementary file 1 — Supplementary file1 (PDF 225 KB) [file 11606_2025_9807_MOESM1_ESM.pdf]


A Project Funded by the Ethics Summer Research Fellowship Program,
Carver College of Medicine, University of Iowa

An Anonymous Survey of Medical Students' Attitudes toward Physicians' Use of Persuasion in Shared Decision Making

This survey has two parts:

Part I: 3 questions based on a case vignette with three scenarios;

Part II: 28 questions which are divided into eight categories.

Definitions used for this survey:

Inform: to impart information or knowledge

Recommend: to suggest an act or course of action as advisable

Persuade: to move by argument to a belief, position, or course of action

Persuasion in healthcare: persuasion is attempted when, after having communicated information and a recommendation, a healthcare professional uses reasoning to try to change a patient's thinking toward a decision that is more consistent with the healthcare professional's understanding of what is good for the patient's health.

Here is the case vignette with three scenarios, each of which is followed by a question:

CASE VIGNETTE: A 60-year-old man has a history of hypercholesterolemia, hypertension, and type 2 diabetes. Five years earlier he moved to a new town but did not, until now, get around to establishing care with a new primary care physician. Having recently turned 60, his health has been on his mind, so he contacted a local clinic.

***Please consider the following 3 scenarios for this patient*:**

Scenario 1: Physician attempts to persuade the patient to exercise regularly by walking

At his first appointment, the primary care physician completes a full history and exam and orders routine lab tests. The physician learns about the patient's lifestyle habits and discusses the importance of healthy eating and regular exercise. The patient says he does not exercise regularly, though he likes to go fishing. The physician informs the patient of the benefits of 30 minutes of aerobic exercise five times per week and recommends exercising regularly by walking. The patient agrees walking is a good idea but says he doesn't enjoy it and is not really interested in exercising, because it's boring and burdensome. On further probing, he acknowledges that he has the time and ability to walk regularly if he wants to, and denies any physical, environmental, safety, or footwear impediments to walking. Based on the patient's response, the physician repeats the recommendation for walking, explaining in more detail the many health benefits it provides, especially for people at greater risk for cardiovascular disease. The patient again says he is not interested in walking for exercise.

Despite the patient declining the recommendation twice, the physician tries a third time to reason with the patient to persuade him to change his mind about walking, this time emphasizing that by not exercising regularly he is putting himself at increased risk of heart disease and early death.

Please respond to the following statement:

**It is ethically appropriate for the physician to attempt to persuade the patient to change his decision about walking by discussing it with him**	Strongly agree Agree Not sure Disagree Strongly disagree

a third time. ❑ ❑ ❑ ❑ ❑

Scenario 2: Physician attempts to persuade the patient to take a statin

At a follow-up appointment two weeks later, the physician calculates the patient's cardiovascular risk assessment (ASCVD) score, revealing he is at "very high" risk of having a heart attack in the next 10 years. The physician explains the meaning of this risk score and recommends the patient take a statin medication to reduce the very high risk of having a major cardiac event. The physician explains how statins work and their possible side effects. The patient declines the recommendation because a good friend of his developed troubling muscle aches while on a statin. The physician acknowledges that muscle complaints are possible side effect of statins but repeats his recommendation, explaining that most people who take statins don't develop any muscle problems and that, in any case, the possibility of side effects can be monitored and the statin discontinued if necessary. The patient still declines the recommendation.

Despite the patient declining the recommendation twice, the physician tries a third time to reason with the patient to persuade him to change his mind about a statin, this time emphasizing that the patient's risk of heart disease is very high and that by refusing to take a statin he is putting himself at increased risk of having a potentially deadly heart attack.

Please respond to the following statement:

	Strongly agree	Agree	Not sure	Disagree	Strongly disagree
**It is ethically appropriate for the physician to attempt to persuade the patient to change his decision about the statin by discussing it with him a third time.**	❑	❑	❑	❑	❑

Scenario 3: Physician attempts to persuade the patient to stay in the hospital

Three months later, the patient develops chest pain and shortness of breath. He calls 911 and is brought to the Emergency Department. Evaluation reveals evidence of an ST-segment elevation myocardial infarction (STEMI) and mild heart failure. A cardiologist is consulted and explains to the patient why cardiac catheterization and coronary angiography with percutaneous coronary intervention (PCI) are needed, and recommends they be done immediately. The patient initially hesitates, but consents to treatment. He is taken to the cardiac catheterization lab and successfully treated with a coronary stent. He is then taken to the ICU for monitoring and further management. His chest pain has now lessened, but he still has shortness of breath. Bedside echocardiography demonstrates a reduced ejection fraction and abnormal regional wall motion, consistent with congestive heart failure in the setting of an acute myocardial infarction.

In the ICU, the patient tells the cardiologist that he wants to go home now that the stent has fixed his problem. The cardiologist explains that his condition is still serious and requires further monitoring and management of his heart and lung function. The cardiologist recommends that the patient stay in the hospital until his condition is stable enough for a safe discharge. The patient listens and states he is willing to stay in the hospital for four more hours. The cardiologist listens and then suggests the patient take some time to rest and also requests permission to return to the patient in three hours to discuss the decision further, and the patient agrees.

Three hours later the cardiologist returns to the patient's room. The patient says he is going to leave the hospital, even if it means going against the cardiologist's advice. The patient says he feels better now and that there were 'some things' he needed to attend to at home which couldn't wait a few days. He promises he will take any necessary medications and follow-up as an outpatient the following week. The cardiologist tries to learn what is so important for the patient to attend to at home. The patient says he doesn't want to talk about it. The cardiologist again voices his recommendation that the patient stay in the hospital. He also confirms the patient's ability to understand, reason, and appreciate the benefits of staying in the hospital and risks of leaving against medical advice. He concludes there is no evidence the patient lacks decision making capacity.

Despite the patient declining the recommendation twice, the cardiologist tries a third time to reason with the patient to persuade him to change his mind about leaving the hospital, this time emphasizing that if the patient refuses to stay in the hospital, he is putting himself at increased risk of a serious medical complication, including the possibility of sudden cardiac death.

Please respond to the following statement:

	Strongly agree	Agree	Not sure	Disagree	Strongly disagree
**It is ethically appropriate for the cardiologist to attempt to persuade the patient to change his decision about leaving the hospital by discussing it with him a third time.**	❑	❑	❑	❑	❑

You will now see 28 questions which are divided into seven categories:

Attitudes toward Shared Decision Making with Patients

Please respond to the following statements:

	Strongly Agree	Agree	Neutral	Disagree	Strongly Disagree
In general, a physician should **inform** a patient about treatment options.	❑	❑	❑	❑	❑
In general, a physician should **make recommendations** to a patient about treatment options.	❑	❑	❑	❑	❑
In general, a physician should **attempt to persuade** a patient when a patient does not accept a recommendation the physician believes is good for the patient's health.	❑	❑	❑	❑	❑
Within the scope of my responsibility as a medical student, I am willing to participate in attempts to persuade patients who decline recommendations made by the healthcare team.	❑	❑	❑	❑	❑
When I am a physician, I expect to feel a responsibility to attempt to persuade patients to change their minds if they decline a treatment which I think would be good for their health.	❑	❑	❑	❑	❑

Observations of Physicians in Clinical Settings

Please respond to the following statements:

I have observed (at least once) a physician attempt to use persuasion ***appropriately*** with a patient or a patient's decision maker.	❑ Yes ❑ No ❑ Unsure
I have observed (at least once) a physician attempt to use persuasion ***inappropriately*** with a patient or a patient's decision maker.	❑ Yes ❑ No ❑ Unsure
I have observed (at least once) a physician who, in my opinion, should have attempted to use persuasion (but did not) when a patient refused a recommended treatment.	❑ Yes ❑ No ❑ Unsure
I have observed at least one physician who was a good role model for how to use persuasion with a patient in a way that is respectful and clear about the intention to influence the patient's decision.	❑ Yes ❑ No ❑ Unsure
I have observed at least one physician try to influence a patient's treatment decision without making it clear to the patient that they were trying to influence the patient's decision.	❑ Yes ❑ No ❑ Unsure

Participation in Persuasion

Please respond to the following statements:

Within the scope of my responsibility as a medical student, I have willingly participated (at least once) in an ***appropriate*** attempt to persuade a patient who was refusing a treatment the healthcare team was recommending.	❑ Yes ❑ No ❑ Unsure
Within the scope of my responsibility as a medical student, I have felt compelled to participate (at least once) in an ***inappropriate*** attempt to persuade a patient who was refusing a treatment the healthcare team was recommending.	❑ Yes ❑ No ❑ Unsure

**Attitudes toward the Four Principles of Biomedical Ethics**<br><br>Please respond to the following statements:	
I think **beneficence** is:	❑ the most important principle of biomedical ethics.<br>❑ one of the most important principles of biomedical ethics.<br>❑ not one of the most important principles of biomedical ethics.
I think **nonmaleficence** is:	❑ the most important principle of biomedical ethics.<br>❑ one of the most important principles of biomedical ethics.<br>❑ not one of the most important principles of biomedical ethics.
I think **autonomy** is:	❑ the most important principle of biomedical ethics.<br>❑ one of the most important principles of biomedical ethics.<br>❑ not one of the most important principles of biomedical ethics.
I think **justice** is:	❑ the most important principle of biomedical ethics.<br>❑ one of the most important principles of biomedical ethics.<br>❑ not one of the most important principles of biomedical ethics.

**Attitudes toward the Four Principles of Biomedical Ethics and Persuasion in Healthcare**<br><br>Please respond to the following statements:					
**All four principles** of bioethics (beneficence, nonmaleficence, autonomy, justice) are **equally important**.	Strongly Agree ❑	Agree ❑	Neutral ❑	Disagree ❑	Strongly Disagree ❑
Persuasion by healthcare professionals is ethically ***inappropriate*** because it **disrespects patient autonomy**.	Strongly Agree ❑	Agree ❑	Neutral ❑	Disagree ❑	Strongly Disagree ❑
Persuasion by healthcare professionals is ethically ***appropriate*** because it **promotes beneficence and nonmaleficence**.	Strongly Agree ❑	Agree ❑	Neutral ❑	Disagree ❑	Strongly Disagree ❑

Attitudes toward Concepts of health

In discussions about healthcare, a distinction can be made between a '**biostatistical' concept of health** (defined objectively in biological and statistical terms as the absence of disease) and a '**well-being' concept of health** (defined subjectively as the ability to realize one's most essential goals).

Please respond to the following statements about concepts of health:

	Strongly Agree	Agree	Neutral	Disagree	Strongly Disagree
Health depends primarily on an individual's objective biological condition.	❑	❑	❑	❑	❑
Health depends primarily on an individual's subjective perception of well-being.	❑	❑	❑	❑	❑

Demographic Information

Please respond to the following questions:

Which of the following best describes where you currently are within the schedule of your pre-clinical work and clinical rotations in medical school? (If you have taken additional time to complete your curriculum, select the answer that best reflects where your progress would place you within the usual requirements of the four-year curriculum.)

❑ Pre-clinical: Summer between M1/M2 years
❑ Clinical: Summer between M2/M3 years
❑ Clinical: Summer between M3/M4 years
❑ MSTP – Pre-clinical
❑ MSTP – Clinical rotation experience (*fewer than* 6 months of clinical rotations)
❑ MSTP – Clinical rotation experience (*more than* 6 months of clinical rotations)

If you were to choose today, which medical specialty do you plan to practice?

❑ Anesthesia
❑ Dermatology
❑ Emergency Medicine
❑ Family Medicine
❑ General Surgery (including General Surgery subspecialties)
❑ Internal Medicine (including Internal Medicine subspecialties)
❑ Neurology
❑ Neurosurgery
❑ Obstetrics/Gynecology
❑ Ophthalmology
❑ Orthopedic Surgery
❑ Otolaryngology
❑ Pathology
❑ Pediatrics
❑ Physical Medicine and Rehabilitation
❑ Plastic & Reconstructive Surgery
❑ Psychiatry
❑ Radiology
❑ Urology
❑ Other {provide text field}

What is your age? {provide numeric field}

❑ prefer not to say

Please indicate your gender:
❑ Female
❑ Male
❑ Transgender
❑ Non-binary/non-conforming
❑ Prefer to self-describe {provide text field}
❑ Prefer not to say

Which of the following describes your race or ethnicity? (Please select all that apply)

❑ American Indian or Alaskan Native
❑ Asian
❑ South Asian
❑ Black or African-American
❑ Caucasian/White
❑ Hispanic, Latino origin
❑ Middle Eastern or North African
❑ Native Hawaiian or other Pacific Islander
❑ Other ethnicity, please specify: {provide text field}
❑ Prefer not to say

Please indicate your state residency status:
❑ In-state (Iowa) resident
❑ Out-of-state resident
❑ other

Final question (optional): Please use this space if there is anything else you would like to tell us about the use of persuasion by physicians in shared decision making, or about any of the questions in this survey.

If there is nothing else you would like to tell us, you do not need to answer this question.

{provide text field}

❖❖❖ THANK YOU for completing this survey. ❖❖❖
